# Literary Effort as a Relaxation for Medical Men

**Published:** 1910-02-26

**Authors:** 


### The Practitioner's Relaxations.

### LITERARY EFFORT AS A RELAXATION FOR MEDICAL MEN.

In the days of the fathers of medicine the alliance with religion had doubtless much to do with the cultivation of literary excellence in medical writings; for the presentation of religious beliefs has always required the most perfect form which literary skill could furnish. There must be, in such representations, telling and easily remembered phrases and sentences, which can comfort their readers in some more heartfelt way than even "that blessed word Mesopotamia." To the earlier ages it was needful for the practitioner of medicine to write in verbal perfection the story of

> His faultless patience, his unyielding will,
> Beautiful gentleness and splendid skill, before he claimed to be recognised as

> Battling with custom, prejudice, disease,
> As once the son of Zeus with Death and Hell.
> (W. E. Henley.)

Of which of the old votaries of medicine could it have been written

> He was happier using the knife than in trying to save the limb. (Tennyson.)

The echo which comes down to us from the past is rather that quoted recently by Lord Rosebery, that the surgeon must have "the hand of a lady and the heart of a lion." The divorcement of hand and heart is sounded by the late Sir William Gull in one of his now historical aphorisms: "Give your intellect without stint to your profession, not your heart."

The charming lectures of Sir Thomas Watson remained as textbooks long after much of their contents had passed out of date. Sir Astley Cooper's word-pictures were the joy of students when they had ceased to be useful at the examination table. But Sir William Gull was right. The clinical thermometer chart has invaded the province of the ward journal and rapidly substituted a dry record for a graphic description. The sphygmograph and the sphygmometer replaced the art by which the observer made clear the significance of the pulse; while details as to facies and decubitus, which were formerly the story of disease or its progress, were soon to give way to bacteriological examinations and blood counts. These were, and were to be, products of the intellect—they replaced the word-pictures of ill-health which could not be written without some appeal to the heart. Is it true that in this all-intellectual struggle, in this rush to be accurate in the detail measured by our aided senses, our memory and heart have been left idle? If so, literary effort as a relaxation for medical men should find a useful place. With our charts and diagrams we are much in the position of the reputed discoverers of the North Pole; we have to explain our observations to the world at large. Sir William Gull recognised this, for in another place he says: "Knowledge of phenomena, sense, knowledge of forces, interpretations, formulations of forces when known so far as to be subject to formulation, tend to forecast and use. The current is running on in a given line; this we hinder or divert by other forces, kindred or added." Can either medicine or surgery resent the hindering or diverting force of literary presentation which claims only pause, an extension of view, and reconstruction? These bring us back from the knowledge which makes us pose as gods to the necessities of humanity.

R. L. Stevenson says: "Science writes of the world as if with the cold finger of a starfish; it is all true, but what is it when compared to the reality of which it discourses?" (*Pan's Pipes.*) We are apt to miss the recognition of the early age at which literary instinct shows itself; it is most surely in the years of infancy. Words, from their very rarity, have a special meaning which can only be crowded out by neglect; phrases, sentences, and verses come back to give expression to intellectual perceptions when the emotions give rein to the tongue or pen, and often with a force which carries conviction.

The literary perception of the adult lies more within the domain of chambers peopled with pictures of memory and belief. It is hard, and it should be hard, to destroy such record as these chambers contain, for they have the value of antiquity, while the most recent addition to our observation is a neophyte. To assign a place to each observation is imperative; but all our household gods cannot be displaced to give it room without due consideration. Is that consideration more wisely culled only from a charted record, to which the intruder is admitted by one sense, than from the mental effort which subjects it to the criticism of all that our memory, our senses, and our judgment can call to our aid? It is true that we are somewhat conservative of the displacement of our mental furniture and have made it a stumbling-block to advance; but are we not more swift than steadfast in these days? If so, the effort towards literary presentation should be useful not only as a relaxation but in the additional security which it gives to our basis of belief. Our old furniture need not crowd our energies out of place: it is only more likely to yield a prepared corner for a new occupant. When we colonised Canada the most prized settlers were the Gaelic-speaking Scotchmen. Their language was much forgotten, but the fact that they had been bi-lingual raised their power to gain the sympathy of the races on the frontier of our civilisation by a mastery of their language and traditions (Dr. John Brownlee). The memories of effort begat effort, the old furniture was replaced by the new and kindly housed, and all without any hindrance to the strenuous work which filled their days. It should be so with us; work we have, and it is all but overwhelming; time we lack, and it is unprocurable; order alone is within our grip. Let us seek it, while we may, in the cultivation of literary form, gathered from others, nourished by ourselves, until

> Thus, swift and steadfast: thus intent and strong;
> While, thus, apart from toil, our souls pursue
> Some high, calm, spheric tune, and prove our work
> The better for the sweetness of our song.
> (E. B. Browning.)

